# A View on Pathogenesis of ≪Vicious Cancer Progression Cycle≫

**DOI:** 10.3389/fonc.2020.00690

**Published:** 2020-04-30

**Authors:** Polina Schwartsburd

**Affiliations:** Institute of Theoretical and Experimental Biophysics, Russian Academy of Sciences, Pushchino, Russia

**Keywords:** cancer biology, glucose, tumor-bearing host, metabolism, stress, vicious cycle

## Abstract

Unrestricted tumor growth requires a permanent supply of glucose that can be obtained from cancer-stimulated hepatic glucose production and/or glucose redirecting from host insulin resistant tissues to cancer cells. This study proposes a mechanism based on metabolic and hormonal changes that may provoke glucose delivery to cancer cells through two interconnected “vicious cycles” whose continuous activity drives cancer progression. As follows from the proposed here feedback model, these “vicious cycles” result from cancer-mediated manipulation of host glucose sensors. The derived conclusions contribute to a better understanding of cancer pathogenesis and identifying potential therapeutic targets.

## Introduction

Cancer is a proliferative disease of multicellular organisms. Similar to parasites, cancer cells manipulate the metabolism of the host organism, thereby receiving a larger portion of glucose than the host cells (1, 2). To prevent glucose starvation dangerous to such glucose-sensitive organs as the brain, the host organism uses glucose sensors capable of maintaining normoglycemia in response to deficient or excess glucose (3). The mechanism of cancer-mediated manipulation of the host glucose metabolism is unknown. In healthy individuals, the blood glucose level is maintained within a narrow range of 60–140 mg/dl by hypothalamus and pancreas glucose sensors (3, 4) that control the release of neurotransmitters and hormones (4, 5). Although the brain weight amounts only to 2% of the body weight, brain cells consume 20% of O_2_ and 60% of the glucose which is their primary fuel, because neurons are highly sensitive to the glucose deficit that can provoke hypoglycemic coma. For coma prevention, the brain and pancreas use glucose sensors that control, regulate and maintain the glucose levels within the optimal range through the regulated release of catabolic hormones, whose action is associated with mobilization of host reserves essential for glucose synthesis in the liver (6). It can be assumed that a similar situation results from the growth of the cancer cell population because these cells display an increased rate of aerobic glycolysis requiring continuous glucose supply from the tumor-bearing host (7). The current study proposes a pathogenic mechanism with a feedback model that explains the preferential glucose delivery to tumor cells by the formation of a ≪vicious cycle≫ where cancer-induced hypoglycemia triggers the chronic activation of the brain and pancreas glucose sensors, thereby stimulating the release of stress hormones crucial for glucose synthesis.

## How Do Cancer Cells Supply Themselves With Host Glucose?

Cancer and brain cells compete for glucose which is their primary fuel. In brain cells, glucose has many critical functions, including ATP synthesis and production of neurotransmitters and structural components of the cell (8). The extracellular glucose concentration in the brain is significantly lower than that in the blood (~2 vs. ~5 mM) (9), which enhances the risk of brain hypoglycemia resulting from fast tumor growth. Unlike most peripheral tissues, brain neurons suffer an irreversible injury after a few minutes of glucose-starvation. The protective mechanism of the brain includes glucose sensors that constantly monitor and improve the glucose level to strictly retain it within the physiological margins. For this purpose, special glucose-sensing neurons and islet α- and β-cells function in a complementary mode. Unlike most neurons using glucose as fuel, the glucose-sensing cells utilize it in a concentration-dependent manner as a signaling molecule to regulate their membrane potential (5, 10). The two types of hypothalamus glucose-sensing cells are excited either by elevating glycemic levels [glucose-excited (GE) neurons] or by a decreasing blood glucose level [glucose-inhibited (GI) neurons]. The GE-neurons can be considered as brain analogs of the islet β-cells, whereas GI-neurons bear some similarity to α-cells (3, 5, 11). It is suggested that these glucose sensors are incorporated into the host monitoring system that recognizes the glucose concentration signal and restores deflected glucose levels to the physiological range (12). The glucose sensors co-work with parasympathetic and sympathetic nerves that control the release of neurotransmitters and hormones, including glucose-lowering insulin and glucose-rising glucagon (13). In brief, the net effect of sympathetic stimulation is an increase in glucagon release and a decrease in insulin release; the opposite response of parasympathetic stimulation was also observed (6).

**Figure 1:**
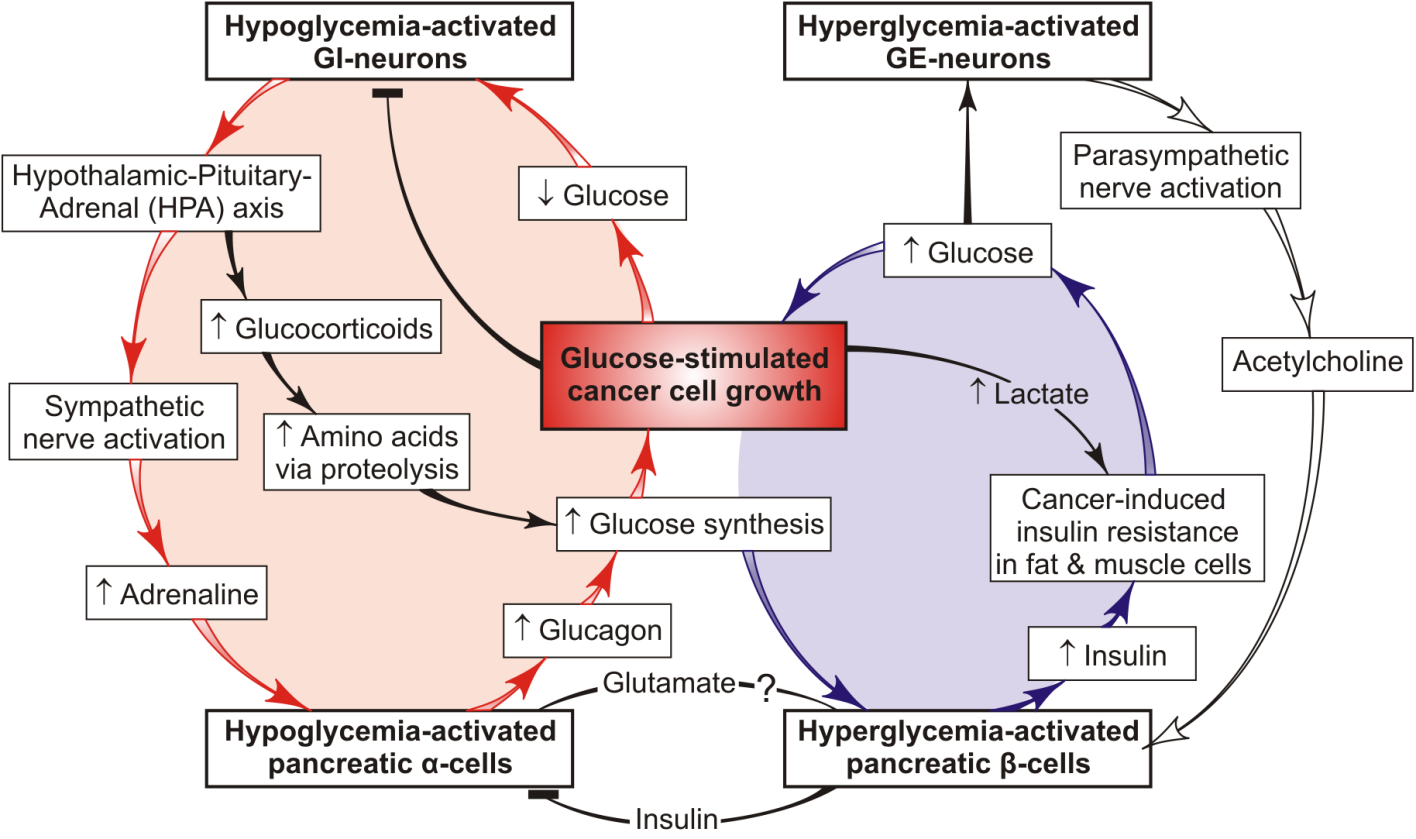
Feedback model of two interconnected tumor-promoting cycles that form a common “vicious cancer progression cycle”. One vicious cycle (red) ensures chronic synthesis of glucose in the liver via the chronic stress-induced mobilization of host reserves and provides preferential glucose supply to cancer cells. This cycle involves cancer-mediated hypoglycemia signals transduced to host glucose sensors that activate the secretion of catabolic hormones (such as adrenaline, glucocorticoids, and glucagon), thus stimulating hepatic glucose production. Persistent signal repeats increase the risk of cancer progression and chronic stress conditions. The hypoglycemia-induced host response to tumor growth may be brain-protecting in the short-run but on a chronic basis, it is dangerous because chronic stress promotes cancer progression and cachexia in the host organism. The other vicious cycle (blue) functions in a complementary mode redirecting the available glucose from host insulin-resistant tissues to cancer cells against the background of the reduced insulin-stimulated glucose uptake into skeletal muscles and adipose tissue (16). This model illustrates how systemic glucose metabolism can be reprogrammed by cancer cells via hormonal deregulation. It also offers a mechanism based on metabolic and hormonal derangements that may favor glucose delivery to cancer cells through a “vicious cancer progression cycle” whose long-term activity drives cancer development and stress conditions in the host organism.

Cancer is a systemic disease implying unrestrained proliferation of cells that continuously consume host glucose through aerobic glycolysis. Cancer cells can up-regulate the low efficiency of aerobic glycolysis via increased glucose consumption from circulation (7), which entails an increased risk of transduction of a hypoglycemia signal to specific glucose sensors functioning solely within the hypoglycemia range. Stimulation of these sensors (e.g., GI-neurons) triggers a cascade of hormone-controlled events, including activation of pancreatic glucose sensors, such as islet α-cells, followed by secretion of glucagon that promotes hepatic gluconeogenesis from non-carbohydrate precursors (14). Glucocorticoids, adrenaline, and glucagon are stress hormones that induce increased hepatic synthesis of glucose, a key substrate of cancer and brain cells. When cancer cells display a higher rate of glucose consumption than brain cells, the signal change-over from hypoglycemia to hyperglycemia may happen again and again, thus forming the vicious cycle of glucose supply to cancer (not host) cells; for the model of this vicious cycle, see Figure 1 (red).

Endogenic glucose is not only consumed by cancer and brain cells but also can serve as a signal to the glucose β-cell sensor stimulating insulin secretion. Yet, it cannot be utilized by fat and muscle cells because they possess cancer-induced insulin resistance (IR) (15, 16). IR is defined by inability of insulin to accomplish its function, specifically in assisting glucose delivery to muscle, fat and liver cells. The previous review (15) is focused on putative effects and mechanisms showing how tumor-host metabolic interactions form the “vicious cycle” (Figure 1, blue) which supports tumor growth via redirecting unutilized glucose from insulin-resistant host tissues to cancer cells. Presumably, the responsible agent is cancer-secreted lactate that is able to reduce extracellular pH and binding affinity between insulin and its receptor, thus provoking the host IR (17). Fatty acids derived from lipolysis in fat cells or lipid droplets (LD) are able to induce the IR (18). Many aggressive cancer cells and cancer microenvironment contain a large number of the LD that have lipolytic enzyme (19), because the cleavage of LD-localized lipid can function as source of fatty acids responsible to induce the host IR. Cancer-increased glucocorticoid levels are also associated with IR (20).

The bidirectional communication between these distinct pathways of the preferential glucose delivery to cancer cells is presented in Figure 1 as two interconnected vicious cycles that form the common “vicious cancer progression cycle.” One of them (red) leads to chronic activation of hepatic glucose synthesis resulting from feedback interactions between tumor cells, host glucose sensors (such as brain GI-neurons, islet α- and β-cells), and the liver. The other (blue) can redirect the glucose supply from the host insulin-resistant tissues to cancer cells. These cycles are complementary and can either restore or increase the blood glucose level, thereby facilitating further tumor growth. On the other hand, the persistent catabolic signals are transduced to the host tissues, thus exhausting the energy resources and impairing the general state of the organism, which provides a basis for cancer progression. This is why the proposed cycle is termed “vicious cancer progression cycle.” It includes a number of events that may serve as potential targets in cancer therapy and/or host protection against cancer progression. It seems to be of great importance to define which of them drive the vicious cycle and what pathways can be used to interfere with its development.

## Potential Anticancer Targets in the Vicious Cycle

Uncontrolled cancer cell proliferation is associated with change-over from oxidative respiration to aerobic glycolysis that requires constant glucose supply, partially through the glucose-delivering vicious cycle (Figure 1); inhibition of glycolytic enzymes is the most important target in cancer treatment (21), along with the inhibition of glucose transport into cancer cells (22, 23). This property of cancer cells modifies the relationship between anabolic and catabolic pathways of glucose metabolism in the host organism and forms the pathological cancer-host vicious cycle (Figure 1) supporting tumor growth. Figure 1 presents a simple feedback model of this process showing the potential therapeutic targets. For example, fasting, calorie restriction and the carbohydrate-restricted ketogenic diet have been successfully used to limit glucose availability and slow cancer progression in a variety of animal models and human studies (24). These dietary manipulations produce a metabolic shift unfavorable for highly glucose-dependent cancer cells because these cells cannot efficiently consume ketone bodies as fuel. As a result, ketones provide retardation of tumor growth and a longer lifetime of mice with metastatic cancer (25). One of the ketone bodies, β/γ-hydroxybutyrate, inhibits glucagon secretion from α-cells in parallel with a decrease in hepatic glucose synthesis (26). Similarly, insulin and somatostatin suppress the production of glucagon and glucose (27), thereby impeding tumor growth. Also, intranasally administered insulin decreases hepatic gluconeogenesis and glucagon secretion, while the anti-diabetic drug metformin antagonizes the glucagon action, thus reducing glucose synthesis (28) and vicious cycle activity. The use of combined therapy (serotonin + tributyrin) is another way to suppress the cancer-driven hypoglycemia in tumor-bearing hosts. A similar effect can be produced by endogenic serotonin or gut-derived butyrate (2). All the above targets are related to the vicious cycle (Figure 1, red) acting under catabolic host conditions, while the insulin resistance-mediated targets are related to the other vicious cycle (Figure 1, blue) analyzed previously (15). However, many respects of the cancer-host metabolic interactions remain obscure, such as the distant impact of cancer cells on the host stress response initiating the formation of the vicious cycles. An example is an application of β-adrenergic antagonists (“β-blockers”) as the therapeutic agents improving the clinical outcome of lung cancer patients (29). Moreover, as epidemiologically evidenced, patients taking β-blockers as anti-arrhythmia and anti-hypertension drugs exhibit a considerably lower susceptibility to several types of cancer (30). These effects can be explained by the ability of cancer cells to activate the sympathetic nervous system and stimulate the release of adrenaline whose interaction with adrenergic receptors of pancreatic α-cells increases glucagon secretion. Because adrenaline-stimulated glucagon, in turn, increases glucose production, the glucose blood level can be lowered through the β-blocker-caused decrease of glucagon concentration. Further investigation of this problem would contribute to the development of cancer therapy and the identification of therapeutic targets.

## Concluding Remark

Similar to parasites, cancer cells depend on their hosts in sustenance and proliferation; they exploit the organism's resources and thereby impair the host's health. For unrestrained growth, these cells must acquire the capacity to instruct the host to grow new blood vessels to constantly provide them with glucose. What pathological mechanism is responsible for this activity? As follows from the analysis presented here, tumor cells can supply themselves with host glucose by creating a glucose-delivering vicious cycle shown as the feedback model in Figure 1. This model contributes to better understanding the metabolic basis of adverse cancer effects on the organism and identifying the potential therapeutic targets. Depending on size, location, and stage of development, tumors produce various effects on the host glucose metabolism and its regulators. Therefore, peculiarities of the metabolism of a certain patient must be taken into account to ensure better cancer therapy. The current paper helps better understand the pathogenesis of cancer progression and identify potential targets that can be used for the selection and/or correction of personalized cancer treatment. In summary, the presented analysis describes the possibility of clinical inhibition of the vicious cycle activity to prevent or improve the catabolic host state associated with tumor growth and progression. However, many questions yet remain to be answered to provide new insight into cancer biology. Specifically, further studies are required to understand in what way tumor cells can remotely reprogram the host's metabolism to their advantage, how this negative impact can be suppressed, and where the therapeutic intervention should be targeted.

## Author Contributions

The author confirms being the sole contributor of this work and has approved it for publication.

## Conflict of Interest

The author declares that the research was conducted in the absence of any commercial or financial relationships that could be construed as a potential conflict of interest.
